# Physiological, Morphological and Antioxidant Responses of *Pediococcus pentosaceus* R1 and *Lactobacillus fermentum* R6 Isolated from Harbin Dry Sausages to Oxidative Stress

**DOI:** 10.3390/foods10061203

**Published:** 2021-05-26

**Authors:** Huan Zhang, Jianhang Xu, Qian Chen, Hui Wang, Baohua Kong

**Affiliations:** College of Food Science, Northeast Agricultural University, Harbin 150030, China; zhanghuan123@neau.edu.cn (H.Z.); xujianhang@neau.edu.cn (J.X.); chenqian@neau.edu.cn (Q.C.); huiwang@neau.edu.cn (H.W.)

**Keywords:** probiotics, *Pediococcus pentosaceus*, *Lactobacillus fermentum*, oxidative stress, cell morphology, antioxidant activity

## Abstract

As functional starter cultures and potential probiotics, the ability of lactic acid bacteria to resist oxidative stress is essential to maintain viability and functional properties. This study investigates the effects of H_2_O_2_ at different concentrations (0, 1, 2, and 3 mM) on the physiological, morphological, and antioxidant properties of *Pediococcus pentosaceus* R1 and *Lactobacillus fermentum* R6 isolated from Harbin dry sausages. The increase in H_2_O_2_ concentration induced a significant increase in reactive oxygen species and a decrease in intracellular ATP levels (*p* < 0.05). Based on scanning electron microscopy, transmission electron microscopy, and electric conductivity analysis, H_2_O_2_ stress caused cell deformation, the destruction of cell membrane integrity, partial loss of the cytoplasm, and an increase in the cell conductivity of both strains. H_2_O_2_ stress with 1 mM or 2 mM concentrations could effectively improve the scavenging rates of free radicals, the activities of superoxide dismutase and glutathione peroxide, and the total antioxidant capacity of both strains (*p* < 0.05). In conclusion, an appropriate oxidative stress contributed to the activation of the antioxidant defense system of both strains, conferred strains a better effect in inhibiting the oxidation of fermented foods, and improved the health of the host.

## 1. Introduction

Lactic acid bacteria (LAB) are innocuous and beneficial microorganisms extensively used in a variety of fermented foods [1,2], and they are widely used in many functional fermented foods as bio-ingredients [3]. LAB play a crucial role in many fermentation processes, as they control the growth of harmful bacteria and improve the sensory quality and texture of foods [4,5]. In addition, some specific strains of LAB have been demonstrated to have antioxidant potential and can capture and neutralize reactive oxygen species (ROS) to delay or prevent oxidation of fermented foods [6,7]. More importantly, many studies have illustrated that some probiotic LAB strains, especially *Lactobacillus*, can eliminate excessive ROS to prevent or alleviate several diseases related to oxidative stress [8,9]. Therefore, LAB with antioxidant potential can be used as functional starters for the fermentation of foods and be involved in probiotic fields to bring health benefits to hosts. 

During production, storage, or application, LAB as a starter culture easily suffer from various environmental stress factors, including osmotic, salt, acid, oxygen, limited fermentation substrate, and low or high temperature.As probiotics, LAB are vulnerable to various stress factors in hosts, such as ROS, bile salts, gastric juice, and intestinal fluid. All these stress factors may affect the activity and physiological function of LAB [10,11,12,13]. Oxidative stress is one of the most common stress factors in bacteria, which usually means the excessive accumulation of ROS [14,15,16]. Due to the lack of a complete electron transport chain and catalases [16,17], LAB are extremely susceptible to the accumulated or acute instant ROS, which can cause the oxidation of cell components, perturb cell activity, and affect the physiological performance and antioxidant activity of LAB [18]. Therefore, the regulation of oxidative stress in LAB cells is vital for maintaining bacterial viability during production, storage, or application. However, the relationship between oxidative stress tolerance and antioxidant capacity in LAB is still not fully understood.

To cope with oxidative stress, LAB have developed a series of adaptive mechanisms, including the production of reducing enzymes, ROS-detoxifying enzymes, small antioxidant molecules, and protein and DNA repair enzymes [17]. The antioxidant properties of LAB are well documented, and various strains show antioxidant capacity. Research has shown that antioxidant enzymes in LAB play crucial roles in developing resistance to different stress factors [19]. Zhao et al. [20] found that hydrogen peroxide (H_2_O_2_)-induced oxidative stress in white-rot fungus, *Coriolus versicolor*, stimulated the activity of intracellular antioxidant enzymes. In the fungus *Aspergillus niger* (B1-D), oxygen enrichment caused reduced ATP yield and elevated endogenous superoxide [21]. However, there are few reports on the effects of oxidative stress on the physiology and antioxidant capacity of LAB and their antioxidant defense systems.

In our previous study, *P. pentosaceus* R1 and *L. fermentum* R6 isolated from Harbin dry sausages showed significant antioxidant potential [4], and they also showed great potential as probiotics [22]. However, little is currently known about the physiological, morphological, and antioxidant properties of LAB under oxidative stress. Thus, the purpose of this trial was to examine the influence of oxidative stress induced by increasing H_2_O_2_ concentrations on the physiological performance of *P. pentosaceus* R1 and *L. fermentum* R6 by measuring biomass, ROS, intracellular ATP levels. Furthermore, the morphological characteristics of both strains were revealed by scanning electron microscopy (SEM), transmission electron microscopy (TEM), and electric conductivity. The antioxidant activities of both strains, after treatment with different H_2_O_2_ concentrations, were assessed by analyzing the free radical scavenging rates, reducing power, the activities of superoxide dismutase (SOD) and glutathione peroxidase (GHS-Px), and total antioxidant capacity (T-AOC).

## 2. Materials and Methods

### 2.1. Bacterial Strains, Growth Conditions and Colony Counts

The *P. pentosaceus* R1 and *L. fermentum* R6 strains were suspended in MRS broth with 25% glycerol and preserved at −80 °C. The activated bacteria were prepared and then inoculated into MRS broth for 12 h (early stationary phase) for the subsequent experiments. The bacteria were collected by centrifugation at 6000× *g* for 10 min at 4 °C and washed three times with sterile phosphate-buffered saline (PBS, 0.1 M, pH 7.4), resuspended in fresh sterile MRS broth containing different H_2_O_2_ concentrations (0, 1, 2, and 3 mM) at a bacterial concentration of 10^8^ CFU/mL, and incubated at 37 °C for 1 h. The cells were collected and washed three times with sterile PBS (0.1 M, pH 7.4), and then the bacterial cells were resuspended in PBS. The effect of H_2_O_2_ treatment on the number of LAB viable cells was assessed with the plate count method.

### 2.2. Intracellular ROS Levels

The formation of intracellular ROS in the two strains was quantified using the peroxide-sensitive fluorescent probe 2′,7′-dichlorodihydrofluoresceindiacetate (H_2_DCFDA, Beyotime Biological Technology Co., Ltd., Shanghai, China) fluorescent detection kit. After treatment with different H_2_O_2_ concentrations, 1.0 mL of cell suspension was washed three times with 0.1 M PBS (pH 7.4), treated with 10 μM H_2_DCFDA, and incubated in the dark for 30 min at 37 °C. Finally, the intracellular fluorescence intensity was assessed at 488/525 nm (excitation/emission) using a fluorescence reader (SpectraMax M2e, Molecular Devices Co., Sunnyvale, CA, USA).

### 2.3. Intracellular ATP Concentration

The intracellular ATP levels in strains were assessed with ATP assay kit (Beyotime Biological Technology Co.). In brief, after three washes with 0.1 M PBS (pH 7.4) solution, the bacteria were lysed with radioimmunoprecipitation assay (RIPA) lysis solution and centrifuged at 12,000× *g* for 10 min at 4 °C. The supernatant was collected for the ATP assay. ATP working reagents (100 μL) were added to a micro-well and left to stand for 5 min. Following this, 100 μL of the supernatant was added to the ATP working reagents. Finally, the luminescence intensity of the mixture was recorded with a luminescent plate reader (Thermo Fisher Scientific Inc., Waltham, MA, USA), and the final ATP concentration was expressed as nmol/mg protein.

### 2.4. Electron Microscopy Analysis

The effects of oxidative stress on the surface and inner morphology of the bacteria were assessed by SEM and TEM using the method as reported by Sanhueza et al. [23] and Ngamdee et al. [24]. All bacteria cells were immersed in 2.5% glutaraldehyde overnight at 4 °C. Then, the cells were treated with 1% osmium tetroxide for 2 h and quickly washed three times with PBS (0.1 M, pH 6.8). Next, step-gradient dehydration was performed with 30%, 50%, 70%, and 90% ethanol concentrations at 15 min intervals; then, the cells were rinsed twice in 100% ethanol for 10 min. For SEM, the sample dehydration procedure was performed gradually with increasing concentrations of ethyl alcohol (50%, 70%, 90%, and 100%). The ethanol was subsequently replaced with hexamethyldisilazane, and the samples were freeze-dried and coated with gold–palladium. Photomicrographs were taken using a Scios Dual Beam SEM (Thermo Fisher, Hillsboro, OR, USA). For TEM, after the samples were dehydrated, they were washed twice in propylene oxide, infiltrated in a 1:1 mixture of epoxy resin and propylene oxide at 37 °C for 1 h, and subsequently infiltrated in a 2:1 mixture of epoxy resin and propylene oxide at 37 °C overnight before being embedded in pure epoxy resin and polymerized at 60 °C overnight. Ultrathin sections were cut, stained, mounted, and observed under a H-800 TEM (Hitachi, Ltd., Tokyo, Japan) at 200 kV.

### 2.5. Electric Conductivity

The conductivity of *P. pentosaceus* R1 and *L. fermentum* R6 suspensions treated with different H_2_O_2_ concentrations was measured every 10 min for 60 min using a conductivity meter (Mettler Toledo FE20/EL20, Shanghai, China) [25].

### 2.6. Assessment of Antioxidant Activity

#### 2.6.1. Intact Cell Suspension and Intracellular Cell-Free Extract Preparation

Bacterial cells treated with different concentrations of H_2_O_2_ were washed three times with PBS, and the cell concentrations were diluted to 10^8^ CFU/mL with PBS. Intact cell suspension was disrupted by ultrasonic cell crusher on ice (JY96-Ⅱ, Xinzhi Biotechnology Co., Ltd., Ningbo, China) and centrifuged at 8000× *g* for 10 min at 4 °C to remove the cell debris, and the supernatant solution was employed as intracellular cell-free extract. The antioxidant activities of the intact cell and intracellular cell-free extract were determined as follows.

#### 2.6.2. 2′,2′-Diphenyl-1-Picrylhydrazyl Radical Scavenging Assay

The 2′,2′-diphenyl-1-picrylhydrazyl (DPPH) radical scavenging rates of the strains were analyzed by following the method of Chen et al. [4]. A 1.0 mL sample was incubated with 2.0 mL DPPH ethanol solution for 30 min in the dark. After centrifugation, the absorbances of the supernatant at 517 nm were recorded. The following formula was used to calculate the DPPH scavenging rates:

Scavenging rates (%) = [1 − (*A*_s_ − *A*_b_)/*A*_c_] × 100% (1)

where *A*_s_ denotes the absorbance of the solution containing samples and DPPH, *A*_b_ denotes the absorbance of the blank (samples and ethanol), and *A*_c_ denotes the absorbance of solution without the samples.

#### 2.6.3. Hydroxyl Radical Scavenging Assay

The hydroxyl radical (•OH) scavenging rates of the strains were determined, and all steps were performed following the method described by Wang et al. [26]. In brief, an aliquot (0.1 mL) of sample PBS (0.2 M, pH 7.4), 1.0 mL of O-phenanthroline (0.1%, *w/v*), 1.0 mL of 2.5 mM FeSO_4_, and 0.5 mL of samples were mixed to obtain a homogeneous mixture; an aliquot (1.0 mL) of 20 mM H_2_O_2_ was reacted with the mixture to trigger the reaction at 37 °C. After 1.5 h, the mixture was centrifuged (10,000× *g*, 10 min), and the absorbance (*A*_s_) of the supernatant was read at 536 nm. The following formula was adopted to calculate the scavenging rates (%):

Scavenging rates (%) = [(*A*_s_ − *A*_0_)/(A − *A*_0_)] × 100% (2)

where *A*_s_ represents the absorbance of the reaction mixture, *A*_0_ represents the absorbance of the reaction mixture without sample, and A represents the absorbance of the reaction mixture in the absence of H_2_O_2_.

#### 2.6.4. Superoxide Radical Scavenging Rates Assay

The superoxide radical (O_2_^•−^) scavenging rates of the strains were analyzed following the method of Liu et al. [27] with a small modification. An aliquot (0.1 mL) of the sample, 2.8 mL of Tris–HCl buffer (0.05 M, pH 8.2), and 0.1 mL of pyrogallic acid (0.05 M) comprised the reaction mixture, and it was left to equilibrate for 4 min at 25 °C and then were neutralized with 1.0 mL HCl (8 M). The reaction mixture absorbance was read at 320 nm. The scavenging rates were calculated according to the following equation:

Scavenging rates (%) = [1 − *A*_sample_/*A*_blank_] × 100% (3)

where *A*_sample_ represents the absorbance of the test sample and *A*_blank_ represents the absorbance of the control reaction without sample.

#### 2.6.5. Reducing Power Assay

The method of Oyaizu [28] was used to assess the reducing power. In total, 0.5 mL of PBS (0.2 M, pH 6.6) and 0.5 mL of potassium ferricyanide (1%, *w/v*) were successively mixed with 0.5 mL of samples. The mixture was rapidly cooled after incubation at 50 °C for 20 min. Following this, 0.5 mL of 10% trichloroacetic acid (*w/v*) was added to the mixed solution and the new mixture was centrifuged at 5000 × *g* for 10 min. Next, the supernatant (1.0 mL) was added to 1.0 mL of ferric chloride (0.1%, *w/v*) and incubated for 10 min. Finally, the absorbance of the resultant mixture was recorded at 700 nm.

#### 2.6.6. Measurement of Antioxidant Enzyme Activities

SOD and GHS-Px activities and the T-AOC of the intracellular cell-free extracts were measured using commercially available assay kits (Nanjing Jiancheng Bioengineering Institute, Nanjing, China). The activities of SOD and GSH-Px were expressed as nmol/mg protein.

### 2.7. Statistical Analysis

All experiment trials were repeated three times, with each test being performed three times. All data were expressed as mean values ± standard errors, and they were evaluated by a general linear model procedure (Statistix 8.1 Analytical Software, St Paul, MN, USA). Statistical differences between different experimental groups were analyzed by one-way analysis of variance (ANOVA) with Tukey’s multiple comparisons.

## 3. Results and Discussion

### 3.1. Survival of the Strains under Oxidative Stress

**Figure 1:**
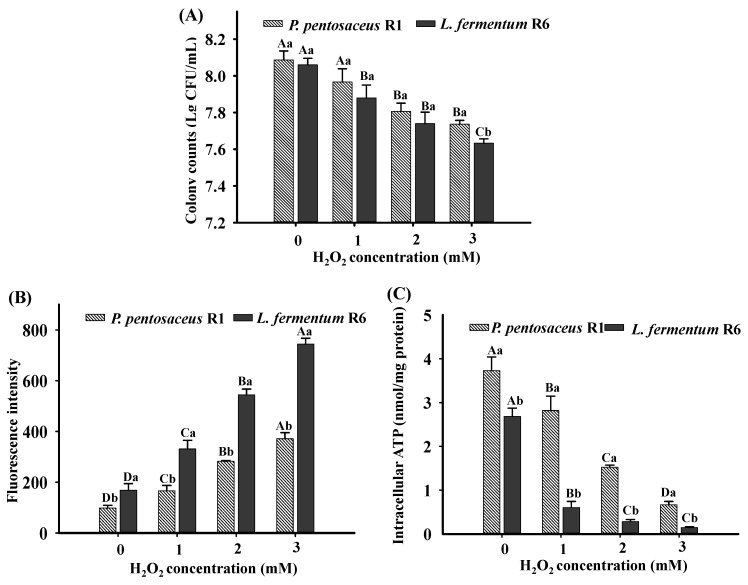
Changes in colony counts (**A**), intracellular reactive oxygen species (ROS) levels (**B**), and intracellular ATP concentrations (**C**) of *Pediococcus pentosaceus* R1 and *Lactobacillus fermentum* R6 incubated in MRS broth with different H_2_O_2_ concentrations for 1 h. Different uppercase letters (A–D) denote statistical differences (*p* < 0.05) within the same strain under different H_2_O_2_ concentrations; different lowercase letters (a and b) denote statistical differences (*p* < 0.05) for both strains within the same H_2_O_2_ concentration.

When LAB are used as functional starters, their resistance to oxidative stress is essential to the production of fermented foods. The colony counts of *P. pentosaceus* R1 and *L. fermentum* R6 were significantly decreased with increasing H_2_O_2_ concentrations (*p* < 0.05; Figure 1A). H_2_O_2_ is the most commonly used oxidizing agent and can generate various ROS in bacteria, resulting in oxidative stress and leading to protein, fat, and DNA damage [29]. Generally, LAB are tolerant to oxidative stresses, as they can scavenge free radicals through their redox system and intracellular antioxidant compounds [16]. However, metabolic disturbance and death of cells can occur when the rate of ROS production exceeds the antioxidant capacity of the LAB [30]. Hence, a decrease in colony counts of both strains compared with the control may be due to the H_2_O_2_-induced excessive ROS production, resulting in the metabolic disturbance and the death of partial cells. In addition, the colony counts of *P. pentosaceus* R1 in the 3 mM H_2_O_2_-treated group showed a significantly higher value than those of *L. fermentum* R6 (*p* < 0.05), which revealed that the tolerance of *P. pentosaceus* R1 to 3 mM H_2_O_2_ was stronger than that of *L. fermentum* R6. A similar result was proved by our previous report by Chen et al. [4], who investigated the antioxidative activities of both strains and found that *P. pentosaceus* R1 showed a higher radical scavenging activity and reducing power than *L. fermentum* R6. As described by Feng and Wang [15], the antioxidant mechanisms of LAB are complex, and different LAB species may rely on different mechanisms of defense against oxidative stress, which may explain why the tolerance of *P. pentosaceus* R1 to H_2_O_2_ was stronger than that of *L. fermentum* R6.

### 3.2. Intracellular ROS Levels

Excessive ROS production can disturb cellular homeostasis, lead to the oxidative damage of cellular components, and induce changes in bacterial growth kinetics and physiological function [31]. To verify the presence of oxidative stress induced by H_2_O_2_, intracellular ROS levels of *P. pentosaceus* R1 and *L. fermentum* R6 in all groups were measured. As shown in (Figure 1B), H_2_O_2_ treatment of both strains resulted in significantly increased ROS levels in an H_2_O_2_ dose-dependent manner (*p* < 0.05), which confirmed that the increased H_2_O_2_ concentrations accelerated intracellular ROS accumulation in both strains. A similar result was also emphasized by Liu et al. [32], who found that the intracellular ROS levels in *Candida oleophila* increased with H_2_O_2_ treatment. Importantly, the increase in ROS levels in both strains treated with H_2_O_2_ compared with the control was reflected in the decreased colony counts of the strains when exposed to oxidative stress, which follows the results illustrated in (Figure 1A). H_2_O_2_ was used as an inducer of oxidative stress, which can react with ferrous iron and produce extremely toxic •OH by the Fenton reaction and cause severe cellular protein damage and break phosphodiester bonds in DNA molecules. The accumulation of oxidation byproducts can react with lipid moieties within the plasma membrane and further induce cell oxidative damage, triggering an oxidation chain reaction and resulting in a rapid accumulation of ROS [15].

### 3.3. Intracellular ATP Concentrations

As a vital representative of energy metabolism, ATP plays an important role in many biological functions, including growth, survival, and replication, and is involved in stress responses [33]. To evaluate whether oxidative stress influences the energy metabolism of the two strains, intracellular ATP concentrations were evaluated. As shown in Figure 1C, ATP concentrations decreased in both strains with increasing H_2_O_2_ concentration. Compared to the control, the ATP levels in *P. pentosaceus* R1 and *L. fermentum* R6 were reduced by about 82% and 95% at 3 mM H_2_O_2_, respectively. As is well established, oxidative stress can cause changes in many biological processes of cells, and ATP is essential in many biological processes as an important energy-supply molecule. It is well-documented that the excess in ROS induced by H_2_O_2_ can severely impair the cell respiratory chain, leading to a decrease in ATP levels [34]. Meanwhile, the ROS, especially extremely toxic •OH, can damage proteins, leading to a lower intracellular energy level [15]. Moreover, Jozefczuk et al. [35] concluded that all three major ATP-production pathways, the metabolism of glycolysis, the pentosephosphate pathway, and the tricarboxylic acid cycle, in *Escherichia coli* suffered from oxidative damage under oxidative stress, leading to a decrease in ATP levels. In addition, oxidative damage repair is an important way by which bacteria deal with oxidative stress, and this process consumes large amounts of ATP [36]. Therefore, the decreased ATP concentrations in both strains under H_2_O_2_ treatment may be ascribed to the oxidative stress induced by H_2_O_2_ leads to the destruction of the ATP production pathways and/or the consumption of ATP in order to repair oxidative damage. 

### 3.4. SEM Analysis

**Figure 2:**
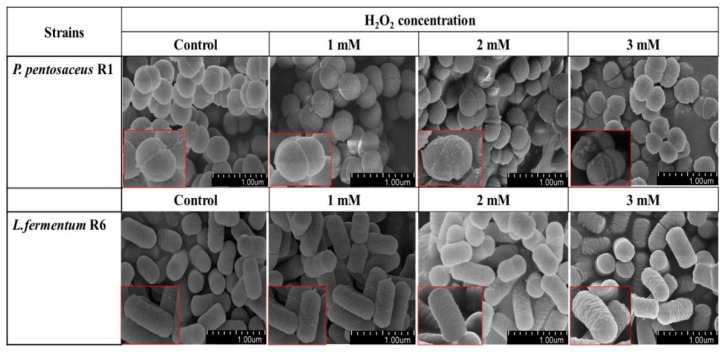
Scanning electron micrographs of *Pediococcus pentosaceus* R1 and *Lactobacillus fermentum* R6 incubated in MRS broth with different H_2_O_2_ concentrations for 1 h.

The SEM images show changes in the cell morphology of the two bacteria after exposure to H_2_O_2_ at different concentrations. Compared with the control, the cell morphology of the two strains showed no obvious change when treated with 1 mM H_2_O_2_. However, when the H_2_O_2_ concentration increased, some particles accumulated on the cell surface of *P. pentosaceus* R1 (Figure 2). According to Gray and Jakob [37], these particles might be inorganic polyphosphates, universally conserved biopolymers with oxidative stress resistance that have multiple functions in increasing bacterial oxidative stress resistance, such as preventing the aggregation of oxidative-damage proteins, reducing free radical concentrations via chelating metal ions, and regulating general stress response pathways in different bacteria. However, the most common ways that bacteria protect themselves against oxidant stress consist in the regulation of enzymatic and non-enzymatic antioxidant defense systems; thus, the protective effect of polyphosphates to *P. pentosaceus* R1 deserves future study. For *L. fermentum* R6, the cell surface changed from smooth to wrinkle with the increase in H_2_O_2_ concentrations from 0 mM to 3 mM. A potential explanation for these results may be that oxidative stress can enhance the expression level of matrix metalloproteinases, which can regulate the degradation of the extracellular matrix proteins, resulting in wrinkles [38]. Gallegos-Monterrosa et al. [39] found that the stress induced by hypoxanthine leads to wrinkle formation in *Bacillus subtilis* 168 and claimed that cell death correlates with wrinkle formation. Based on the information of this entire paragraph, it is tempting to conclude that the *P. pentosaceus* R1 presents a more positive defense performance than *L. fermentum* R6, which may further explain why the tolerance of *P. pentosaceus* R1 to H_2_O_2_ was stronger than that of *L. fermentum* R6 as illustrated in Figure 1A.

### 3.5. TEM Analysis

**Figure 3:**
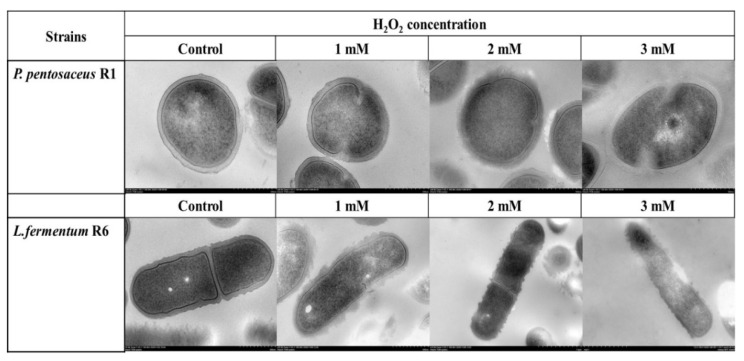
Transmission electron micrographs of *Pediococcus pentosaceus* R1 and *Lactobacillus fermentum* R6 incubated in MRS broth with different H_2_O_2_ concentrations for 1 h.

TEM was applied to detect the microstructure and more intuitively observe morphological changes of the bacteria after exposure to different concentrations of H_2_O_2_. As shown in (Figure 3), the intracellular organization of both control strains exhibited good integrity and even distribution. However, cell membrane integrity was gradually destroyed and cell cytoplasm was partially lost on H_2_O_2_ treatment. At a higher H_2_O_2_ concentration (3 mM), we observed elongation of the *P. pentosaceus* R1 and *L. fermentum* R6 cells, and the *L. fermentum* R6 cell elongation was gradually aggravated in an H_2_O_2_-concentration-dependent manner. A similar result was illustrated by Guo et al. [40], who found that NaCl stress results in cell elongation for *Escherichia coli* and concluded that an appropriate elongation of bacterial cells is beneficial in enhancing resistance to adverse conditions and that bacteria are capable of rapidly reverting to normal cell morphology when the adverse environmental factors are removed. According to Kijima et al. [41] and Mattick et al. [42], the elongation of bacteria cells under stress suppresses cell division and⁄or DNA synthesis process, which was characterized by avoiding the consumption of ATP to ensure the intracellular ATP was involved in the defense against stress. Therefore, modest changes in morphology are considered an adaptation strategy of bacterial cells to the stressful environment [43].

### 3.6. Extracellular Electrolytes

**Figure 4:**
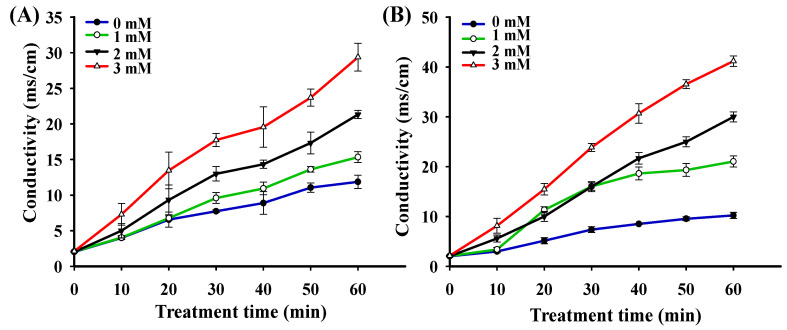
Analysis of electric conductivity of *Pediococcus pentosaceus* R1 (**A**) and *Lactobacillus fermentum* R6 (**B**) treated with different H_2_O_2_ concentrations for 1 h.

When the cell membrane is destroyed by the external environment, it will cause a large number of electrolytes to undergo exosmosis, leading to an increase in conductivity; therefore, the evolution of membrane conductivity can be considered to be an indicator of membrane integrity [44]. To observe the impact of H_2_O_2_ stress on the membrane integrity of both strains, the electric conductivity of cell suspensions was measured, as shown in (Figure 4). H_2_O_2_ stress compromised the cell membrane integrity of both strains, as the conductivity of both strains gradually increased with the increase in H_2_O_2_ concentration (*p* < 0.05). This observation is consistent with the TEM images presented in (Figure 3), in which it is apparent that the degree of cell membrane damage is positively correlated with H_2_O_2_ concentration. In addition, the conductivity of *P. pentosaceus* R1 suspension was lower than that of *L. fermentum* R6 suspension after treatment with the same H_2_O_2_ concentrations for 60 min, which indicates that the membrane damage degree of *P. pentosaceus* R1 was lower than that of *L. fermentum* R6 under the same oxidative stress conditions. This conclusion was consistent with the TEM images observed in Figure 3, which further confirms that the tolerance of *P. pentosaceus* R1 to H_2_O_2_ was stronger than that of *L. fermentum* R6. The bacterial cell envelopes are the first line of defense against stressors for bacteria. When excess ROS is present, the cell envelope stress response is also triggered, and the major components of the bacterial cell wall and cell membrane, i.e., peptidoglycans, surface layer proteins, and phospholipids, are vulnerable, threatening the integrity and viability of the cells [43,45]. 

### 3.7. Free Radical Scavenging Rates and Reducing Power

**Figure 5:**
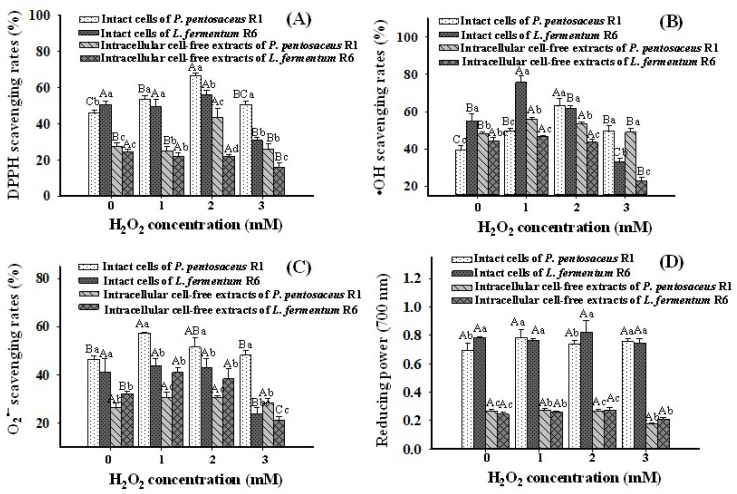
Changes in 2′,2′-diphenyl-1-picrylhydrazyl (DPPH) radical scavenging rates (%) (**A**), hydroxyl radical (•OH) scavenging rates (%) (**B**), superoxide anion radical (O_2_^•−^) scavenging rates (%) (**C**), and reducing powers (**D**) of *Pediococcus pentosaceus* R1 and *Lactobacillus fermentum* R6 after incubation in MRS broth with different H_2_O_2_ concentrations for 1 h. Different uppercase letters (A–C) denote statistical differences (*p* < 0.05) of the same components in the same strain (intact cell or intracellular cell-free extract) under different H_2_O_2_ concentrations; different lowercase letters (a–d) denote statistical differences (*p* < 0.05) for both strains within the same H_2_O_2_ concentration.

The antioxidant ability of both strains treated with different H_2_O_2_ concentrations was assessed by measuring the scavenging rates of DPPH radical, •OH radical, and O_2_^•−^ radical, and reducing power. As shown in (Figure 5A), the scavenging rates of DPPH radical in *P. pentosaceus* R1 increased with increasing H_2_O_2_ concentrations from 0 mM to 2 mM and then decreased at 3 mM H_2_O_2_ for both intact cells and intracellular cell-free extractions. For *L. fermentum* R6, the DPPH radical scavenging rates at 1 mM and 2 mM H_2_O_2_ showed no significant difference (*p* > 0.05) compared with the control but decreased at 3 mM H_2_O_2_ (*p* < 0.05) for both intact cells and intracellular cell-free extractions. As shown in (Figure 5B), the •OH radical scavenging rates of intact cells in *P. pentosaceus* R1 treated with all concentrations of H_2_O_2_ were significantly higher than that of the control, and intact cells at 2 mM H_2_O_2_ expressed the maximum scavenging rates (*p* < 0.05); for the intracellular cell-free extraction in *P. pentosaceus* R1, treatment with 1 mM and 2 mM of H_2_O_2_ resulted in significantly higher •OH scavenging rates that seen in the control (*p* < 0.05). For *L. fermentum* R6, intact cells at 1 mM H_2_O_2_ demonstrated the maximum •OH scavenging rates (*p* < 0.05), which then decreased with increased H_2_O_2_ concentration. The •OH scavenging rates of the intracellular cell-free extraction of *L. fermentum* R6 at 1 mM and 2 mM H_2_O_2_ were not significantly different from the control (*p* > 0.05). This is consistent with the results reported by Amanatidou et al. [46], who claimed that •OH scavenging levels were similar in *Lactobacillus sake* cells exposed to anaerobic and high O_2_ conditions and proposed that the total level of •OH scavenging of bacteria may be an endogenous property of bacteria and is not regulated by the imposed oxidative stress level. However, the rate at 3 mM H_2_O_2_ was significantly lower than the control (*p* < 0.05) in our results, which may be ascribed to 3 mM H_2_O_2_ inducing intracellular damage, resulting in decreased •OH scavenging ability of *L. fermentum* R6. The O_2_^•−^ scavenging rates of intact cells *of P. pentosaceus* R1 and the intracellular cell-free extraction of *L. fermentum* R6 increased at 1 mM and 2 mM H_2_O_2_ and then decreased at 3 mM H_2_O_2_ (*p* < 0.05) (Figure 5C). The O_2_^•−^ scavenging rates of intact cells of *L. fermentum* R6 decreased at 3 mM H_2_O_2_ compared with the control. Unexpectedly, the reducing power of both strains showed no significant change with H_2_O_2_ treatment (*p* < 0.05), as shown in (Figure 5D), and the reducing powers of the intact cells of both strains were significantly higher than those of the intracellular cell-free extractions (*p* < 0.05). 

In most cases, the ability of the two strains to scavenge various free radicals was improved at 1 mM and 2 mM H_2_O_2_ and decreased at 3 mM H_2_O_2_, possibly because the relatively low H_2_O_2_ levels induced the antioxidant defense system, improving antioxidant performance, while 3 mM H_2_O_2_ led to a disruption of the overall metabolic status of the cells and a decrease in free radical scavenging rates [47]. 

### 3.8. Antioxidant Enzyme Activities Analysis

**Figure 6:**
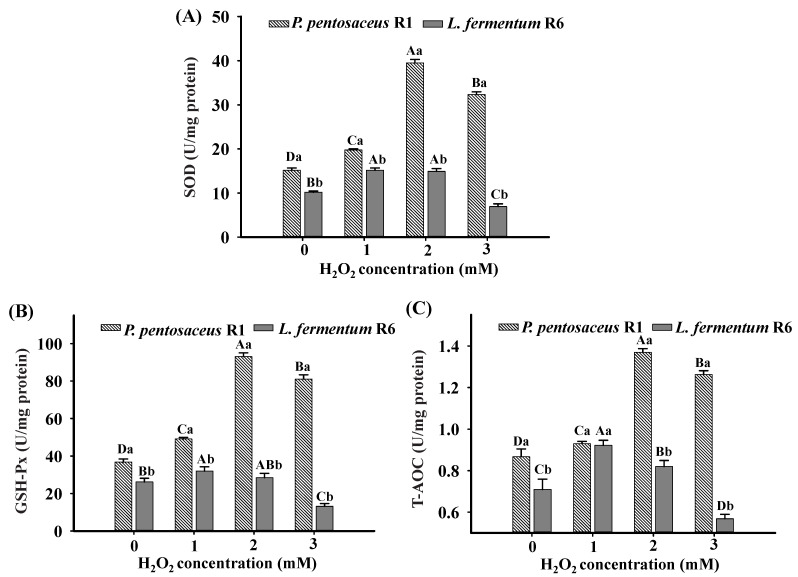
Superoxide dismutase (SOD) activity (**A**), glutathione peroxide (GSH-Px) activity (**B**), and total antioxidant capacity (T-AOC) (U/mg protein) (**C**) of *Pediococcus pentosaceus* R1 and *Lactobacillus fermentum* R6 after incubation in MRS broth with different H_2_O_2_ concentrations for 1 h. Different uppercase letters (A–D) denote statistical differences (*p* < 0.05) within the same strain under different H_2_O_2_ concentrations; different lowercase letters (a and b) denote statistical differences (*p* < 0.05) for both strains within the same H_2_O_2_ concentration.

The SOD and GSH-Px play pivotal roles in the antioxidant defense in cells [48]. Many studies have proven that antioxidant enzymes can act as cell defenders by scavenging ROS [49]. To identify the influence of oxidation on antioxidant capacity, we investigated the SOD and GSH-Px activities and the T-AOC of both strains treated with various H_2_O_2_ concentrations. As shown in Figure 6, H_2_O_2_ treatment notably increased the SOD and GSH-Px activities and increased the T-AOC in *P. pentosaceus* R1, and the highest SOD and GSH-Px activity levels and T-AOC were observed at 2 mM H_2_O_2_ (*p* < 0.05). The overall activities of SOD and GSH-Px and the T-AOC were drastically reduced in *L. fermentum* R6 than in *P. pentosaceus* R1 (*p* < 0.05). These results are in accordance with the phenomenon observed in (Figure 1), confirming that the tolerance of *P. pentosaceus* R1 to oxidative stress was stronger than that of *L. fermentum* R6. In addition, H_2_O_2_ concentrations of 1 mM and 2 mM resulted in increased SOD and GSH-Px activities and an increase in the T-AOC in *L. fermentum* R6, while 3 mM H_2_O_2_ induced relatively lower SOD and GSH-Px activities and T-AOC. A similar conclusion was also illustrated by Zhang et al. [45], who investigated the effect of mesotrione-induced oxidative stress on the antioxidant enzyme activities of *Chlorella vulgaris*. They found that SOD and CAT activities increased upon exposure to 18 mg/L mesotrione, while 37.5 mg/L mesotrione resulted in decreased antioxidant enzyme activities and speculated that a high dose of the mesotrione directly inhibited the antioxidant enzyme activities. Meanwhile, our results demonstrated that the ability of the two strains to scavenge various free radicals was effectively improved by enhancing the activities of antioxidant enzymes under oxidative stress. For *L. fermentum* R6, 3 mM H_2_O_2_ may have caused severe oxidative damage that eventually resulted in significant metabolic perturbations and decreased antioxidant enzyme activity [46].

## 4. Conclusions

This study indicated that H_2_O_2_ induced oxidative stress in a dose-dependent manner which seriously affects cell homeostasis, leading to LAB cells’ partial death and reduces the intracellular ATP levels. Meanwhile, oxidative stress changed the morphology and reduced the membrane integrity of *P. pentosaceus* R1 and *L. fermentum* R6. The *P. pentosaceus* R1 displayed a stronger defense property than *L. fermentum* R6, which corresponded to the higher tolerance to H_2_O_2_ and the better antioxidant enzyme activities of *P. pentosaceus* R1 than that of *L. fermentum* R6. In addition, relatively appropriate oxidative stress levels (1 and 2 Mm H_2_O_2_) triggered antioxidant enzymatic defense system and improved the antioxidant activities of *P. pentosaceus* R1 and *L. fermentum* R6. Notably, *P. pentosaceus* R1 has better antioxidant activity than *L. fermentum* R6 under some oxidative stress conditions. Overall, *P. pentosaceus* R1 had better potential than *L. fermentum* R6 for application as an antioxidant starter in fermented food systems and as probiotic in the functional foods or host’s gastrointestinal tract when the cells are exposed to aerobic food or host systems. 

## Data Availability

The date presented in this study are available in the article.
